# Association between Polymorphisms in Vascular Endothelial Growth Factor Gene and Response to Chemotherapies in Colorectal Cancer: A Meta-Analysis

**DOI:** 10.1371/journal.pone.0126619

**Published:** 2015-05-08

**Authors:** Lei Wang, Shan Ji, Zeneng Cheng

**Affiliations:** Research Institute of Drug Metabolism and Pharmacokinetics, School of Pharmaceutical Sciences, Central South University, Changsha, Hunan, China; University General Hospital of Heraklion and Laboratory of Tumor Cell Biology, School of Medicine, University of Crete, GREECE

## Abstract

**Background:**

Some studies have investigated the effects of polymorphisms in the vascular endothelial growth factor (VEGF) gene on responsiveness to chemotherapy for colorectal cancer (CRC) and have shown inconclusive results.

**Methods:**

Eligible studies that assessed the associations between polymorphisms in the VEGF gene and response to chemotherapy in CRC were searched in the PubMed, Embase and Medline databases until November 2014. Odds ratios (OR) and 95% confidence intervals (CIs) were used to evaluate the associations, using Review Manager software, version 5.3. Stratified analysis was also conducted.

**Results:**

In the overall analysis, a significant association with responsiveness to chemotherapy in CRC was identified in CC vs. CA of the VEGF -2578 C/A polymorphism (OR = 1.40, 95% CI 1.00-1.97, P = 0.05) and in CC+CT vs. TT of the VEGF -460 C/T polymorphism (OR = 0.71, 95% CI 0.53-0.96, P = 0.02). In subgroup analysis, a significant association was found in excluding anti-angiogenic agent subgroup in three comparison models of the VEGF -2578 C/A polymorphism and another three genetic models of the VEGF -460 C/T C/A polymorphism.

**Conclusions:**

CC vs. CA of the VEGF -2578 C/A polymorphism and CC+CT vs. TT of the VEGF -460 C/T polymorphism might be predictive factors of responsiveness to chemotherapy in CRC. However, single-nucleotide polymorphisms in the VEGF gene lacked sufficient predictive ability to determine whether patients with CRC should add anti-angiogenic agents to their chemotherapy regimens.

## Introduction

Colorectal cancer (CRC) is one of the leading causes of death worldwide, and approximately 1 million people are diagnosed with CRC every year [1–2]. It is an enormous challenge to determine the appropriate treatment to improve the poor prognosis of CRC, and the median survival in patients remains less than initially desired [3].

Currently, chemotherapy is widely used in malignant tumors for significant improvements in overall survival (OS) and progression free survival (PFS) in patients [4–5]. Regarding CRC, XELOX (capecitabine + oxaliplatin), FOLFIRI (fluorouracil + leucovorin + irinotecan) and FOLFOX-4 (fluorouracil + leucovorin + oxaliplatin) are all first-line chemotherapy regimens in clinical practice [6]. Recently, new biological therapies employing anti-angiogenic agents, including inhibitors of vascular endothelial growth factor (VEGF) and epidermal growth factor receptor (EGFR), have been combined with the existing chemotherapy regimens [7–8]. The addition of anti-angiogenic agents to first-line chemotherapy regimens has shown efficacy in CRC by significantly prolonging PFS and OS [9]. However, there have been inter-individual differences in the clinical outcomes of patients receiving chemotherapy for CRC. A reliable marker contributes to improving therapeutic outcomes and limiting potential adverse events through identifying patients who will benefit from these therapies.

The VEGF gene is located on chromosome 6p21.3, and its coding region spans approximately 14 kilobases and consists of 8 exons [10–11]. The VEGF gene is highly polymorphic, and numerous single nucleotide polymorphisms (SNPs) have been found in its promoter and 5'-, and 3'- untranslated regions (UTR). VEGF -2578 C/A (rs699947), -460C/T (rs3025039), +405G/C (rs2010963), and +936C/T (rs833061) were the most common SNPs in the VEGF gene, where -2578 C/A and -460C/T were in the promoter, +405G/C was in the 5'- UTR, and +936C/T was in the 3'- UTR. These SNPs have been reported to be associated with variations in VEGF protein production. For example, VEGF -460C/T influences VEGF protein translation efficiency, and VEGF +936C/T affects VEGF expression in tumor tissue [12–13].

CRC is a complicated disease affected by both genetic polymorphisms and environmental factors [14–15]. VEGF gene polymorphisms have been reported to be associated with CRC through regulation of the expression of VEGF, which has been identified as playing a key role in a series of pathologic processes involved in tumor growth and metastasis. Moreover, VEGF-involved angiogenesis pathways are also important targets of chemotherapeutic treatment in CRC [16]. Therefore, VEGF gene polymorphisms have been suggested to influence the response to chemotherapy in CRC, and they might be of great value as potential biomarkers to predict clinical outcomes.

SNPs in the VEGF gene, including -2578 C/A, -460C/T, +405G/C, and +936C/T, have been focused in the relationship of the gene with the response to chemotherapy in CRC [17–24]. However, these studies showed inconclusive results, probably because the sample size included in any single study was so small that it lacked inadequate evidence to demonstrate a comprehensive conclusion. In contrast, meta-analysis is a powerful method for synthesizing information from varied investigations on the same issue [25]. Therefore, a meta-analysis of all eligible studies could provide reliable information about the associations between VEGF polymorphisms and response to chemotherapy in CRC.

In our work, a meta-analysis of all published studies was performed to investigate whether VEGF polymorphisms were associated with responsiveness to chemotherapy in patients with CRC. Moreover, a subgroup analysis with regard to a combination of anti-angiogenic agents in chemotherapy regimens was also performed, to investigate whether SNPs in the VEGF gene could work as biomarkers to predict the outcomes of adding anti-angiogenic agents to chemotherapies for CRC. As far as we know, this was the first systemic review and meta-analysis that focused on the associations between VEGF gene polymorphisms and response to chemotherapy for CRC.

## Methods

### Literature search

All studies assessing the associations between polymorphisms in the VEGF gene and response to chemotherapy in CRC were retrieved via an exhaust search of databases, including PubMed, Embase and Medline. The bibliographic search was performed by two investigators using the following retrieval terms: ("vascular endothelial growth factor a"[MeSH Terms] OR "vascular endothelial growth factor a"[All Fields] OR "vegf"[All Fields]) AND ("polymorphism, genetic"[MeSH Terms] OR ("polymorphism"[All Fields] AND "genetic"[All Fields]) OR "genetic polymorphism"[All Fields] OR "polymorphism"[All Fields])) AND (response[All Fields] OR (clinical[All Fields] AND outcome[All Fields])) AND ("colorectal neoplasms"[MeSH Terms] OR ("colorectal"[All Fields] AND "neoplasms"[All Fields]) OR "colorectal neoplasms"[All Fields] OR ("colorectal"[All Fields] AND "cancer"[All Fields]) OR "colorectal cancer"[All Fields]. Other potentially eligible studies were found by manually searching relevant reviews and the included studies. All of the records were updated to November 2014. Only English-language articles were used for this meta-analysis.

### Inclusion and exclusion criteria

For inclusion in the meta-analysis, studies had to meet the following criteria: (1) studies assessing the associations between polymorphisms in the VEGF gene and response to chemotherapy in CRC; (2) independent prospective or retrospective association studies; and (3) studies providing detailed data to estimate odds ratios (ORs) and corresponding 95% confidence intervals (CIs). Studies were excluded if they were other types of original studies, such as reviews, meta-analyses and case reports. Moreover, studies were also not eligible for this meta-analysis if they lacked critical information.

### Data extraction

The data extraction was conducted independently by two investigators (L. Wang and S. Ji). Inter-researcher discrepancies were settled by discussion or by a third reviewer (Z. N. Cheng). The following critical data were extracted from each eligible study: first author, publication year, ethnicity, number of patients, median age, variation category, treatment modality, response criteria and genotype data.

### Statistical analysis

ORs and 95% CIs were used to evaluate the associations of VEGF gene polymorphisms with response to chemotherapy in CRC, using Review Manager software, version 5.3 (provided by the Cochrane Collaboration), and statistical significance of the OR was ascertained with a P value from the Z-test less than 0.05. Six contrasts for the VEGF -2578 C/A polymorphism were evaluated: comparison of the A allele with C allele; comparison of CC+CA vs. AA; comparison of CC vs. CA+AA; comparison of CC vs. AA; comparison of CC vs. CA; and comparison of CA vs. AA. An evaluation of similar comparison models was also performed in the other three VEGF gene polymorphisms, including VEGF -460 C/T, VEGF +405 G/C and VEGF +936 C/T. Applicability to the effects models depended on the degree of between-study heterogeneity, which was estimated by Cochran's Q test and the I^2^ test in this meta-analysis. The heterogeneity across studies was identified by a significant Q test (P<0.10) or by I^2^>50%; thus, the random effects model was selected for the evaluation of each investigation with combined ORs. In contrast, the fixed effects model was used for P>0.10 from Q test or for I^2^<50%. Subgroup analysis was undertaken of combinations of anti-angiogenic agents in chemotherapy regimens. To evaluate the stability of the outcomes, sequential exclusion of individual studies was performed in the sensitivity analysis [26]. The effect of potential publication bias was assessed with both visual assessment of Begg’s funnel plot and Egger’s test [27]. Two sided P-values were used for statistical decisions in this meta-analysis, and statistical significance was considered when the P-value was less than 0.05.

## Results

### Study characteristics

**Fig 1:**
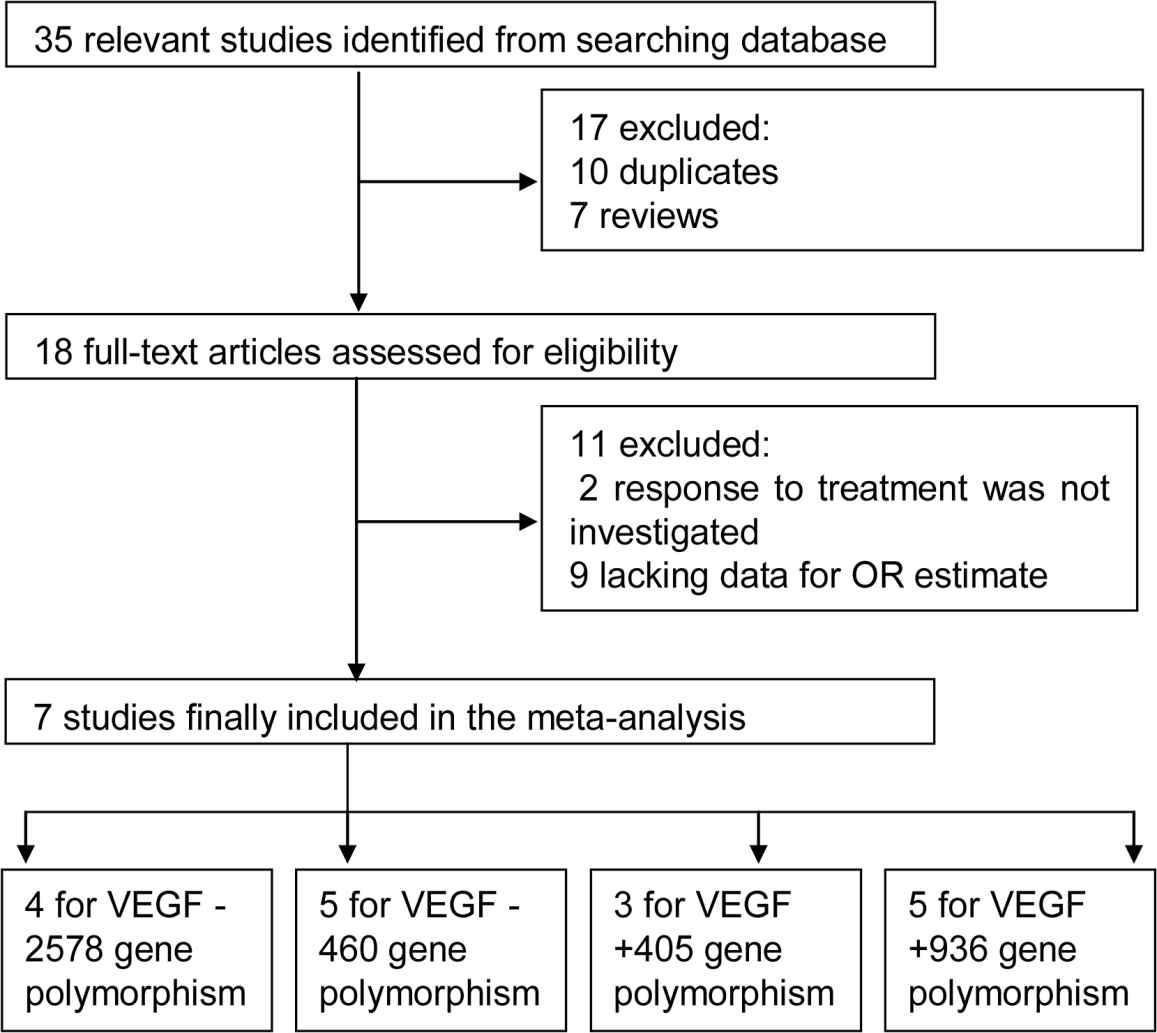
Flow diagram for study selection in meta-analysis.

A total of 35 relevant studies were retrieved from an initial search of the PubMed, Embase and Medline databases. After removing duplicates and reviews, 18 full-text articles were assessed for eligibility. Finally, 7 studies met the inclusion criteria and were included in the meta-analysis [17–23]. Study selection is illustrated in Fig 1.

The characteristics of the selected studies are listed in Table 1. All 7 studies, involving a total of 1184 patients, were included in the meta-analysis, including 4 studies of the VEGF -2578 C/T polymorphism, 5 studies of VEGF -460 C/T, 3 studies of VEGF +405 G/C, and 5 studies of VEGF +936 C/T. The publication years of all of the selected studies ranged from 2006 to 2013.

**Table 1:** Characteristics of the eligible studies considered in this report.

Study (year)	Number of cases	Ethnicity	Age, Median year	SNPs investigated	Treatment protocol	Response criteria
Zhang (2006)	39	Mixed	64	VEGF +936 C/T	Cetuximab	A reduction of at least 50% tumor burden on computed tomography
Lurje (2008)	130	Mixed	NG	VEGF +936 C/T	Cetuximab	At least a 50% reduction in the sum of the bidimensional products of all measurable lesions documented at least 4 wk apart
Chen (2011)	128	Asian	NG	VEGF -460 C/T	FOLFOX-4	RECIST criteria
Hansen (2011)	72	Caucasian	62	VEGF -2578 C/A, VEGF -460 C/T, VEGF +405 G/C, VEGF +936 C/T	XELOX	RECIST criteria
Hansen (2012)	218	Caucasian	62	VEGF -2578 C/A, VEGF -460 C/T, VEGF +405 G/C, VEGF +936 C/T	Chemotherapy-Bev	RECIST criteria
Koutras (2012)	173	Caucasian	64	VEGF -2578 C/A, VEGF -460 C/T, VEGF +405 G/C, VEGF +936 C/T	XELIRI-Bev or FOLFIRI-Bev	RECIST criteria
Loupakis (2013)	424	Caucasian	NG	VEGF -2578 C/A, VEGF -460 T/C	FOLFIRI-Bev	RECIST criteria

Bev: Bevacizumab; FOLFIRI: Fluorouracil + Leucovorin + Irinotecan; FOLFOX-4: Fluorouracil + Leucovorin + Oxaliplatin; NG: Not given; RECIST: Response Evaluation Criteria In Solid Tumors; SNPs: single-nucleotide polymorphisms; XELIRI: Irinotecan + Capecitabine; XELOX: Capecitabine + Oxaliplatin.

### Association of the VEGF -2578 C/A polymorphism with response to chemotherapy in CRC

**Fig 2:**
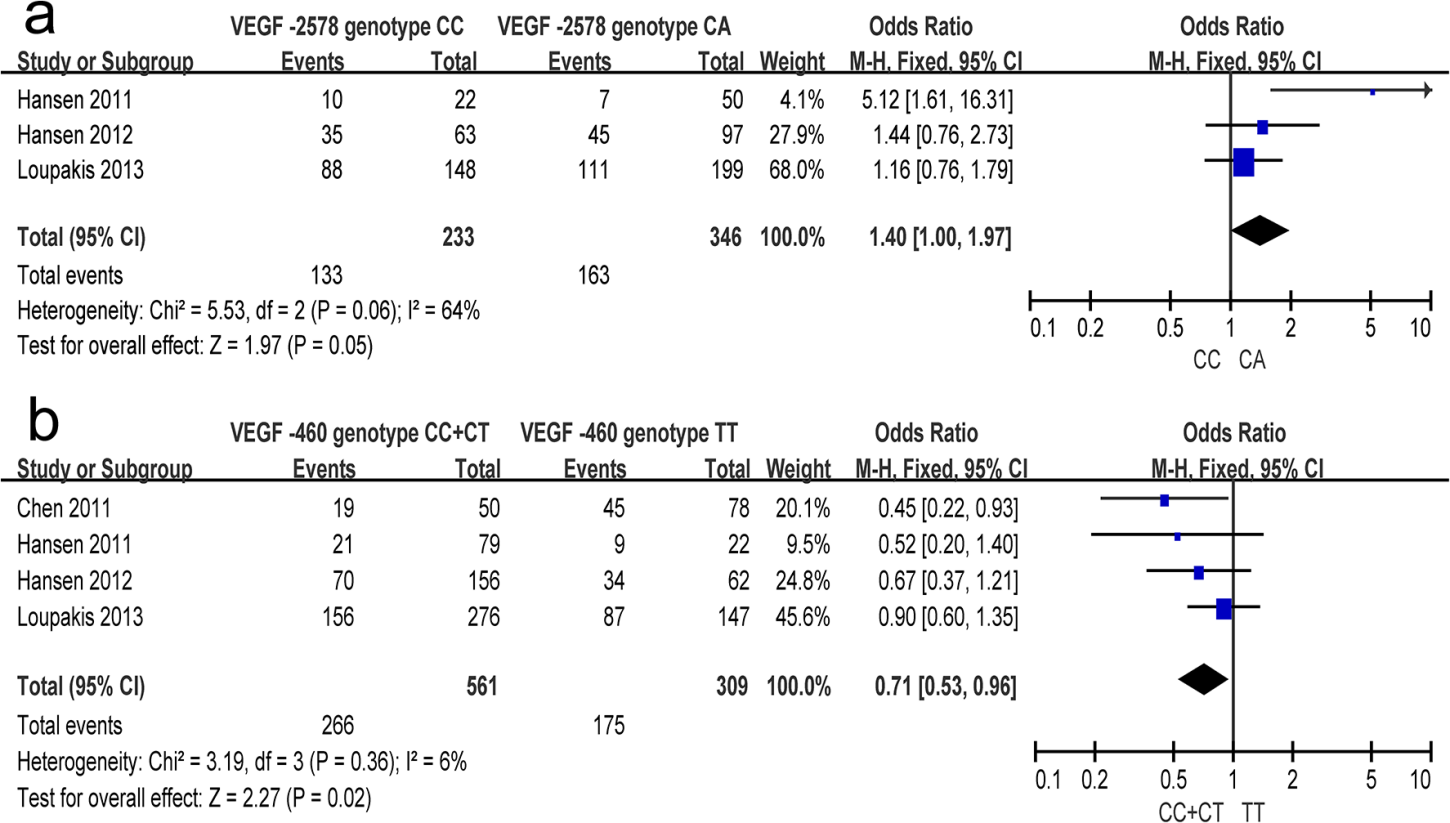
Meta-analysis of the VEGF -2578 C/A polymorphism (a) and VEGF -460 C/T polymorphism (b) with response to chemotherapies in colorectal cancer. (a) Analytical results of the genetic model of CC vs. CA in VEGF -2578 C/A polymorphism. (b) Results in the meta-analysis of the comparison model of CC+CT vs. TT in VEGF -460 C/T polymorphism.

**Fig 3:**
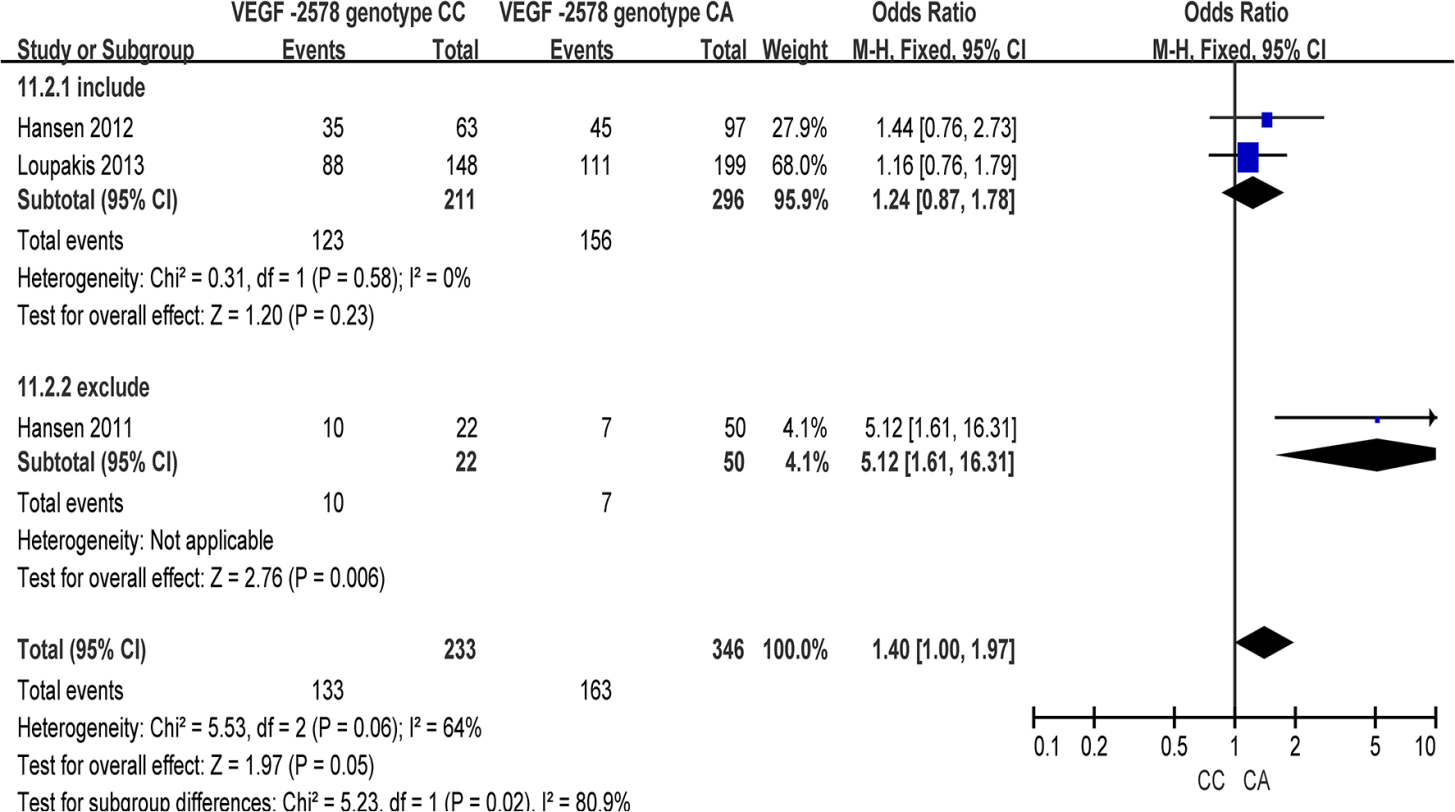
Subgroup analysis of the association between VEGF -2578 C/A polymorphisms with response to chemotherapies in colorectal cancer (CC vs. CA).

A total of 4 studies were included in the meta-analysis. In overall analysis, between-study heterogeneity was apparent in the comparison models of CA vs. AA (I^2^ = 75%, P _heterogeneity_ = 0.02), so a random-effects model was used. However, no evidence of heterogeneity was found in the other five comparison models of the C allele vs. A allele (I^2^ = 0%, P _heterogeneity_ = 0.41), CC vs. CA+AA (I^2^ = 17%, P _heterogeneity_ = 0.30), CC+CA vs. AA (I^2^ = 50%, P _heterogeneity_ = 0.11), CC vs. AA (I^2^ = 0%, P _heterogeneity_ = 0.49), or CC vs. CA (I^2^ = 64%, P _heterogeneity_ = 0.06), so the fixed-effects model was applied for those genetic models (Table 2). A significant association between the VEGF -2578 C/A polymorphism and responsiveness to chemotherapy was found in the comparison model of CC vs. CA (OR = 1.40, 95% CI 1.00–1.97, P = 0.05) (Fig 2A). Moreover, in the subgroup of excluding anti-angiogenic agents, a significant association with responsiveness to chemotherapy was found in three comparison models, including CC+CA vs. AA (OR = 0.38, 95% CI 0.15–0.95, P = 0.04), CC vs. CA (OR = 5.12, 95% CI 1.61–16.31, P = 0.006) (Fig 3) and CA vs. AA (OR = 0.20, 95% CI 0.07–0.59, P = 0.004) (Table 3).

**Table 2:** Meta-analysis of the association between VEGF polymorphisms and response to chemotherapies in CRC.

Polymorphism	Comparison model	Test of heterogeneity		Effects model	Test of association		
		I^2^	P-value		OR	95%CI	P-value
VEGF -2578 C/A	C vs. A	0%	0.41	Fixed model	1.12	0.91–1.38	0.29
	CC vs. CA+AA	17%	0.30	Fixed model	1.34	0.97–1.83	0.07
	CC+CA vs. AA	50%	0.11	Fixed model	1.01	0.74–1.38	0.96
	CC vs. AA	0%	0.49	Fixed model	1.24	0.82–1.86	0.31
	CC vs. CA	64%	0.06	Fixed model	1.40	1.00–1.97	0.05
	CA vs. AA	75%	0.02	Random model	0.68	0.29–1.57	0.37
VEGF -460 C/T	C vs. T	0%	0.44	Fixed model	0.90	0.73–1.11	0.33
	CC vs. CT+TT	23%	0.27	Fixed model	1.02	0.75–1.41	0.88
	CC+CT vs. TT	6%	0.36	Fixed mode	0.71	0.53–0.96	0.02
	CC vs. TT	0%	0.51	Fixed model	0.82	0.55–1.24	0.35
	CC vs. CT	60%	0.08	Fixed model	1.14	0.78–1.67	0.49
	CT vs. TT	28%	0.25	Fixed model	0.76	0.54–1.06	0.11
VEGF +405 G/C	G vs. C	81%	0.02	Random model	1.12	0.46–2.73	0.81
	GG vs. GC+CC	71%	0.03	Random model	1.01	0.50–2.07	0.97
	GG+GC vs. CC	0%	0.37	Fixed model	0.77	0.38–1.57	0.47
	GG vs. CC	59%	0.12	Fixed model	0.81	0.39–1.70	0.58
	GG vs. GC	81%	0.02	Random model	1.36	0.38–4.86	0.63
	GC vs. CC	0%	0.96	Fixed model	0.72	0.33–1.57	0.41
VEGF +936 C/T	C vs. T	0%	0.62	Fixed model	0.81	0.56–1.17	0.26
	CC vs. CT+TT	0%	0.90	Fixed model	0.76	0.54–1.09	0.13
	CC+CT vs. TT	0%	0.49	Fixed model	0.94	0.31–2.91	0.92
	CC vs. TT	0%	0.50	Fixed model	0.86	0.28–2.68	0.80
	CC vs. CT	0%	0.85	Fixed model	0.74	0.48–1.16	0.19
	CT vs. TT	0%	0.48	Fixed model	1.15	0.36–3.70	0.82

OR: odd ratio; CI: confidence interval.

**Table 3:** Subgroup analyses of the association between VEGF polymorphisms and response to chemotherapies in CRC.

Polymorphism	Comparison model	anti-angiogenetic monoclonal antibody drugs	OR	95%CI	P-value
VEGF -2578 C/A	C vs. A	include	1.15	0.92–1.43	0.22
		exclude	0.92	0.50–1.68	0.78
	CC vs. CA+AA	include	1.25	0.90–1.75	0.19
		exclude	2.46	0.92–6.55	0.07
	CC+CA vs. AA	include	1.15	0.82–1.62	0.41
		exclude	0.38	0.15–0.95	0.04
	CC vs. AA	include	1.27	0.82–1.98	0.28
		exclude	1.03	0.34–3.12	0.96
	CC vs. CA	include	1.24	0.87–1.78	0.23
		exclude	5.12	1.61–16.31	0.006
	CA vs. AA	include	1.02	0.67–1.54	0.94
		exclude	0.20	0.07–0.59	0.004
VEGF -460 C/T	C vs. T	include	0.88	0.70–1.10	0.25
		exclude	1.09	0.60–2.00	0.78
	CC vs. CT+TT	include	0.93	0.66–1.30	0.67
		exclude	2.12	0.85–5.27	0.11
	CC+CT vs. TT	include	0.82	0.58–1.14	0.24
		exclude	0.47	0.26–0.85	0.01
	CC vs. TT	include	0.80	0.51–1.24	0.31
		exclude	1.02	0.33–3.14	0.97
	CC vs. CT	include	0.97	0.64–1.46	0.89
		exclude	3.22	1.14–9.03	0.03
	CT vs. TT	include	0.82	0.58–1.18	0.29
		exclude	0.32	0.10–0.97	0.04
VEGF +936 C/T	C vs. T	include	0.86	0.56–1.32	0.49
		exclude	0.66	0.32–1.38	0.27
	CC vs. CT+TT	include	0.80	0.54–1.18	0.26
		exclude	0.59	0.24–1.42	0.24
	CC+CT vs. TT	include	0.85	0.23–3.13	0.81
		exclude	1.28	0.13–12.82	0.83
	CC vs. TT	include	0.81	0.22–3.00	0.76
		exclude	1.04	0.10–10.69	0.97
	CC vs. CT	include	0.82	0.49–1.36	0.43
		exclude	0.55	0.22–1.36	0.20
	CT vs. TT	include	0.96	0.24–3.75	0.95
		exclude	1.89	0.18–20.39	0.60

OR: odd ratio; CI: confidence interval.

### Association of the VEGF -460 C/T polymorphism with response to chemotherapy in CRC

**Fig 4:**
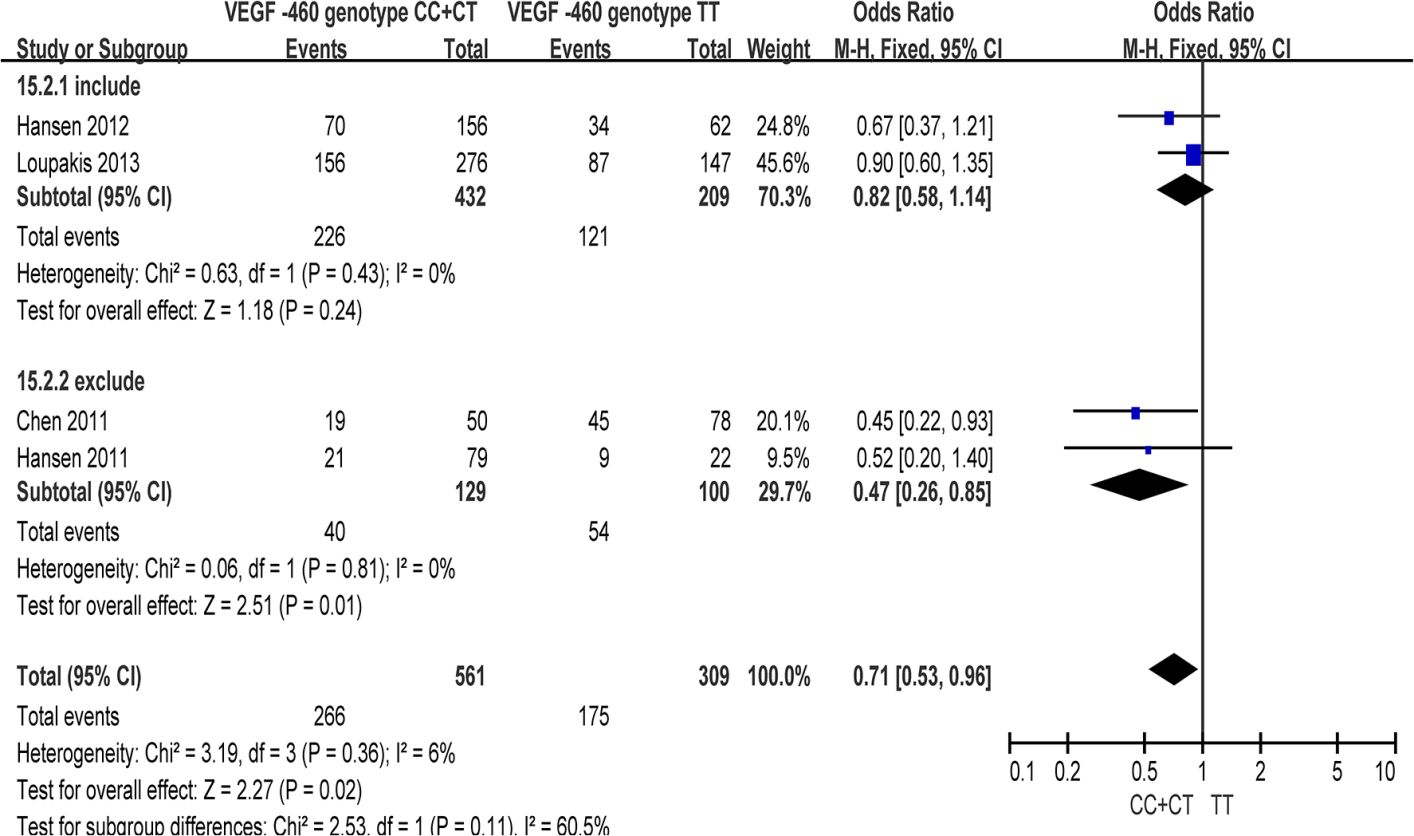
Subgroup analysis of the association between VEGF -460 C/T polymorphisms with response to chemotherapies in colorectal cancer (CC +CT vs. TT).

Five studies were finally included in the meta-analysis. There was no apparent between-study heterogeneity found among the six genetic models, so fixed-effects models were used for all of them (Table 2). In the overall analysis, the VEGF -460 C/T polymorphism was found to be associated with responsiveness to chemotherapy in the comparison model of CC+CT vs. TT (OR = 0.71, 95% CI 0.53–0.96, P = 0.02) (Fig 2B). Moreover, similar results were obtained in the subgroup analysis (Table 3). In the subgroup of excluding anti-angiogenic agents, a significant association between the VEGF -460 C/A polymorphism with responsiveness to chemotherapy was identified in three comparison models, including CC+CT vs. TT (OR = 0.47, 95% CI 0.26–0.85, P = 0.01) (Fig 4), CC vs. CT (OR = 3.22, 95% CI 1.14–9.03, P = 0.03), and CT vs. TT (OR = 0.32, 95% CI 0.10–0.97, P = 0.04).

### Association of the VEGF +405 G/C polymorphism with response to chemotherapy in CRC

This meta-analysis included 5 eligible studies of the association of the VEGF +405 G/C polymorphism with response to chemotherapy in CRC. Due to between-study heterogeneity was found in the three associated comparison models of the G allele vs. C allele (I^2^ = 81%, P _heterogeneity_ = 0.02), GG vs. GC+CC (I^2^ = 71%, P _heterogeneity_ = 0.03), and GG vs. GC (I^2^ = 81%, P _heterogeneity_ = 0.02), random-effects models were utilized. The remaining genetic models all used fixed-effects models. In the overall analysis, no significant associations were found in any of the comparison models including the G allele vs. the C allele (OR = 1.12, 95% CI 0.46–2.73, P = 0.81), GG vs. GC+CC (OR = 1.01, 95% CI 0.50–2.07, P = 0.97), GG+GC vs. CC (OR = 0.77, 95% CI 0.38–1.57, P = 0.47), GG vs. CC (OR = 0.81, 95% CI 0.39–1.70, P = 0.58), GG vs. GC (OR = 1.36, 95% CI 0.38–4.86, P = 0.63), or GC vs. CC (OR = 0.72, 95% CI 0.33–1.57, P = 0.41) (Table 2). In addition, similar results were obtained in the subgroup analysis, with no associations identified in either subgroup including or excluding anti-angiogenic agents (Table 3).

### Association of the VEGF +936 C/T polymorphism and response with chemotherapy in CRC

A total of 4 studies were included in this meta-analysis. The effects model was finally selected as the fixed-effects model for all six comparison models, mainly because no between-study heterogeneity was found in these genetic models. No significant associations between the VEGF +936 C/T polymorphism and response to chemotherapy in CRC were identified in any comparison models, including C allele vs. T allele (OR = 0.81, 95% CI 0.56–1.17, P = 0.26), CC vs. CT+TT (OR = 0.76, 95% CI 0.54–1.09, P = 0.81), CC+CT vs. TT (OR = 0.94, 95% CI 0.31–2.91, P = 0.92), CC vs. TT (OR = 0.86, 95% CI 0.28–2.68, P = 0.80), CC vs. CT (OR = 0.74, 95% CI 0.48–1.16, P = 0.19), or CT vs. TT (OR = 1.15, 95% CI 0.36–3.70, P = 0.82) (Table 2). Additionally, similar results were also obtained in subgroup analysis, with no associations identified in either subgroup including or excluding anti-angiogenic agents (Table 3).

### Sensitivity analysis and publication bias

**Fig 5:**
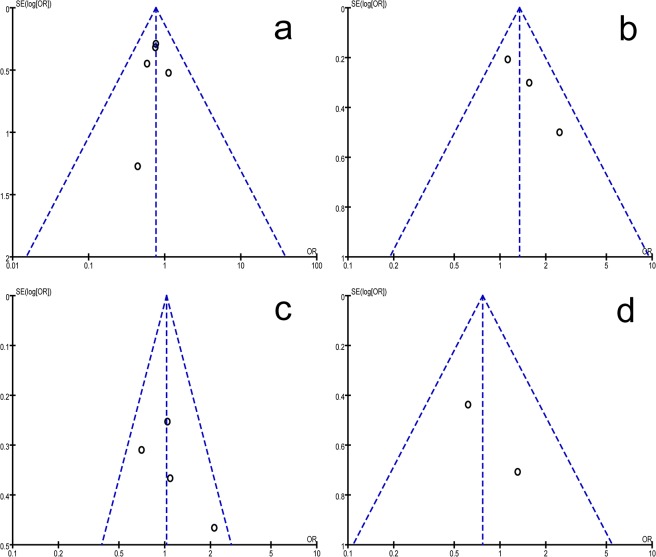
Funnel plots of studies included in the meta-analysis. (a) Funnel plot of the genetic model of CC vs. CT+TT in VEGF + 936 C/T polymorphism. (b) Funnel plot of the comparison model of CC vs. CA+AA in VEGF -2578 C/T polymorphism. (c) Funnel plot of the CC vs. CT+TT model in VEGF -460 C/T polymorphism. (d) Funnel plot of the GG+GC vs. CC model in VEGF +405 C/T polymorphism.

Individual studies were consecutively excluded in the sensitivity analysis to investigate whether the obtained results were robust. The analysis showed that the results obtained in the meta-analysis were statistically robust, because the corresponding combined ORs in all of the separate subgroup analyses were relatively stable when deleting any individual study. Publication bias was evaluated with both visual assessment of Begg’s funnel plot and Egger’s test in the meta-analysis. As illustrated in Fig 5, symmetrical funnel plots indicated that there was no evidence of publication bias for the meta-analysis, and the results of Begg’s test also resulted in the same conclusion (detailed data not show).

## Discussion

Some published studies have reported inconclusive results about the associations between polymorphisms in the VEGF gene and response to chemotherapy in CRC, probably due to limited predictive ability with relatively small sample sizes. For this reason, a meta-analysis was performed to obtain a comprehensive conclusion on the basis of pooled data from all 7 eligible studies. In this meta-analysis, 4 common SNPs in the VEGF gene were systematically investigated for their associations with response to chemotherapy in CRC. As described in Table 2, significant association were found in the CC vs. CA model of the VEGF -2578 C/A polymorphism and the CC+CT vs. TT model of the VEGF -460 C/T polymorphism. However, no significant associations were identified in other models of these two polymorphisms, and similar results were encountered in all of the comparison models of the VEGF +405 G/C and VEGF +936 C/T polymorphisms. Although the number of relevant studies included in the separate analysis was not sufficiently large, valuable evidence was nevertheless provided by synthesizing all of the published data, proving that the CC vs. CA model of the VEGF -2578 C/A polymorphism and the CC+CT vs. TT model of the VEGF -460 C/T polymorphism might be predictive factors to responsiveness to chemotherapy in CRC.

Recently, new biological therapies employing anti-angiogenic agents, including EGFR and VEGF inhibitors, such as cetuximab and bevacizumab, respectively, have been combined with existing chemotherapy regimens because the optimal first-line treatment is no longer chemotherapy alone but a combination with new biological therapies [28]. Therefore, to investigate whether SNPs of the VEGF gene will influence the responsiveness to chemotherapy of patients with CRC through a combination of anti-angiogenic agents, a subgroup analysis was performed subsequently of a combination of anti-angiogenic agents in chemotherapy strategies. Due to the lack of sufficient data to perform a meta-analysis accordingly, the association between the VEGF +405 G/C polymorphism and responsiveness to chemotherapy was not included in the subgroup analysis. On the basis of the results of the subgroup analysis, a significant association of excluding the anti-angiogenic agents subgroup was found in the comparison models of the VEGF -2578 C/A polymorphism, including the CC+CA vs. AA, CC vs. CA, and CA vs. AA models. Additionally, similar results were also obtained for the VEGF -460 C/A polymorphism. In the subgroup analysis, all positive conclusions arose from the subgroup of excluding the anti-angiogenic agents. No associations of VEGF polymorphisms with responsiveness to chemotherapy were found in the subgroup including anti-angiogenic agent, indicating that SNPs in the VEGF gene might have weak ability to predict the responsiveness to chemotherapy of CRC patients receiving anti-angiogenic agents, alone or in combination with other first-line chemotherapy regimens.

Moreover, a significant association with responsiveness to chemotherapy in CRC was identified in the CC vs. CA model of the VEGF -2578 C/A polymorphism and the CC+CT vs. TT model of the VEGF -460 C/T polymorphism. By undertaking subgroup analyses with regard to combinations of anti-angiogenic agents in chemotherapy strategies, we found that associations were only significant in the subgroups excluding anti-angiogenic agents, while negative results were shown in subgroups of including the anti-angiogenic agents (Figs 3 and 4). Therefore, the results indicated that the associations between VEGF polymorphisms and responsiveness to chemotherapy were not derived from receiving anti-angiogenic agents alone or by combining anti-angiogenic agents with other first-line chemotherapy regimens. In other words, although VEGF is a commonly used target of new biological therapies that aim to block the angiogenic pathway, there was limited evidence that the SNPs in the VEGF gene lack sufficient predictive ability as biomarkers, only from the perspective of chemotherapeutic responsiveness, to identify whether patients with CRC should add anti-angiogenic agents to their chemotherapy regimens.

Although surprising but valuable information was initially obtained in this meta-analysis, this meta-analysis was nevertheless limited due to some deficiencies. First, the limited numbers of both the studies and subjects might have provided insufficient statistical power to evaluate the associations between VEGF polymorphisms and responsiveness to chemotherapy. Second, the heterogeneity of chemotherapeutic regimens might have affected the accuracy of the analysis results. Although the limited number of related studies made it difficult to perform a meta-analysis in the present study when stratified according to chemotherapeutic regimens, a more accurate stratification should be undertaken in the future on the basis of more related studies being published. Third, the sources of inter-study heterogeneity could not be addressed for most of the polymorphisms. Fourth, although there was no evident publication bias identified, potential bias might have distorted the results of the meta-analysis. Finally, relevant effects caused by other environmental factors were difficult to estimate due to publication limitations or incomplete raw data.

Although the above limitations existed, this initial meta-analysis of the association between VEGF polymorphisms and responsiveness to chemotherapy in CRC was statistically more persuading than any single study. It concluded that the CC vs. CA model of the VEGF -2578 C/A polymorphism and the CC+CT vs. TT model of the VEGF -460 C/T polymorphism might be predictive factors in responsiveness to chemotherapy in CRC. However, SNPs in the VEGF gene lack sufficient predictive ability as biomarkers to identify whether patients with CRC should add anti-angiogenic agents to their chemotherapy regimes. To assess more accurately the associations between VEGF polymorphisms and responsiveness to chemotherapy in CRC, further studies conducted in standardized and unbiased manner are required.

## Supporting Information

S1 File PRISMA Checklist. (DOC)

S2 File Meta-analysis on Genetic Association Studies Checklist. (DOC)

S3 File List of full-text excluded articles. (DOC)
