# Supplementary material for: Association between Polymorphisms in Vascular Endothelial Growth Factor Gene and Response to Chemotherapies in Colorectal Cancer: A Meta-Analysis
Source: PLoS One. 2015 May 8;10(5):e0126619. doi: 10.1371/journal.pone.0126619 (PMC4425504; doi:10.1371/journal.pone.0126619)
Supplement: S1 File — (DOC) [file pone.0126619.s001.doc]

| **Section/topic** | **#** | **Checklist item** | **Reported on page #** |
| --- | --- | --- | --- |
| **TITLE** | | |  |
| Title | 1 | Association between polymorphism in vascular endothelial growth factor gene and response to chemotherapies in colorectal cancer: a meta-analysis | Title, Page 1 |
| **ABSTRACT** | | |  |
| Structured summary | 2 | Background: Some studies had investigated the effects of polymorphisms in vascular endothelial growth factor (VEGF) gene on responsiveness of chemotherapies in colorectal cancer (CRC), and showed inconclusive results.  Methods: Eligible studies were searched from Pubmed, Embase and Medline databases until November, 2014. Odds ratio (OR) and 95% confidence interval (95% CI) were used to evaluate the associations by using Review Manager 5.3 software. Stratified analysis was also conducted.  Results: In overall analysis, a significant association with responsiveness of chemotherapies in CRC has been identified in the CC vs. CA of VEGF -2578 C/A polymorphism (OR=1.40, 95% CI 1.00-1.97, P=0.05) and CC+CT vs. TT of VEGF -460 C/T polymorphism (OR=0.71, 95% CI 0.53-0.96, P=0.02). In subgroup analysis, a significant association was found in excluding anti-angiogenetic agents subgroup in three comparison models of VEGF -2578 C/A polymorphism, and other three genetic models of VEGF -460 C/T C/A polymorphism.  Conclusions: CC vs. CA of VEGF -2578 C/A polymorphism and CC+CT vs. TT of VEGF -460 C/T polymorphism might be predictive factors to responsiveness of chemotherapies in CRC. However, single-nucleotide polymorphisms in VEGF gene lacked enough predictive ability to identify whether patients with CRC should add anti-angiogenetic agents into their chemotherapy regimes. | Abstract, Page 2 |
| **INTRODUCTION** | | |  |
| Rationale | 3 | Several single-nucleotide polymorphisms (SNPs) in VEGF gene including -2578 C/A, -460C/T, +405G/C, and +936C/T, have been focused on its relationship with the response to chemotherapies in CRC [11-17]. However, these studies showed inconclusive results, probably because the sample size enclosed in any single study is so small that it lacked inadequate evidence to demonstrate a comprehensive conclusion. (References presented in the article) | Introduction, Page 3 |
| Objectives | 4 | In our work, a meta-analysis of all published studies was performed to investigate whether VEGF polymorphisms are associated with responsiveness to chemotherapies in patients with CRC. | Introduction, Page 3 |
| **METHODS** | | |  |
| Protocol and registration | 5 | All studies assessing the associations between polymorphisms in VEGF gene and response to chemotherapies in CRC were retrieved via an exhaust search of databases including Pubmed, Embase and Medline. | Methods, Page 3-4 |
| Eligibility criteria | 6 | For an inclusion in the meta-analysis, studies had to follow such criteria: (1) studies assessing the association between polymorphisms in VEGF gene and response to chemotherapies in CRC; (2) independent prospective or retrospective association studies; (3) provided detailed data to estimate odds ratios (ORs) and according 95% confidence intervals (CIs). | Methods, Page 4 |
| Information sources | 7 | All studies assessing the associations between polymorphisms in VEGF gene and response to chemotherapies in CRC were retrieved via an exhaust search of databases including Pubmed, Embase and Medline. | Methods, Page 3-4 |
| Search | 8 | The bibliographic search was made by two investigators using the retrieve terms as following: ("vascular endothelial growth factor a"[MeSH Terms] OR "vascular endothelial growth factor a"[All Fields] OR "vegf"[All Fields]) AND ("polymorphism, genetic"[MeSH Terms] OR ("polymorphism"[All Fields] AND "genetic"[All Fields]) OR "genetic polymorphism"[All Fields] OR "polymorphism"[All Fields])) AND (response[All Fields] OR (clinical[All Fields] AND outcome[All Fields])) AND ("colorectal neoplasms"[MeSH Terms] OR ("colorectal"[All Fields] AND "neoplasms"[All Fields]) OR "colorectal neoplasms"[All Fields] OR ("colorectal"[All Fields] AND "cancer"[All Fields]) OR "colorectal cancer"[All Fields]. | Methods, Page 4 |
| Study selection | 9 | Two individual investigators (LW & SJ) evaluated the references respectively. Decision for inclusion was made on consensus or by a third reviewer (ZNC). For an inclusion in the meta-analysis, studies had to follow such criteria: (1) studies assessing the association between polymorphisms in VEGF gene and response to chemotherapies in CRC; (2) independent prospective or retrospective association studies; (3) provided detailed data to estimate odds ratios (ORs) and according 95% confidence intervals (CIs). | Methods, Page 4 |
| Data collection process | 10 | Data extraction was conducted independently by two investigators (L. Wang and S. Ji). Inter-researcher discrepancies were settled by discussion or by a third reviewer (Z. N. Cheng). | Methods, Page 4 |
| Data items | 11 | Critical data was extracted from each eligible study: first author, publication year, ethnicity, number of patients, median age, variation category, treatment modality, response criteria and genotype data. | Methods, Page 4 |
| Risk of bias in individual studies | 12 | The χ2 test was performed to appraise Hardy-Weinberg equilibrium (HWE) in the controls. | Methods, Page 4 |
| Summary measures | 13 | ORs and 95% CIs were used to evaluate the associations of VEGF gene polymorphisms with response to chemotherapies in CRC. | Methods, Page 4 |
| Synthesis of results | 14 | The statistical significance of OR was ascertained with Z-test, and P<0.05 was deemed to statistically significant. Applying the fixed-effects model or random-effects model depended on the degree of heterogeneity among studies. The Cochran's Q test was used to estimate between-study heterogeneities, and I2 test was utilized to quantify the effect of heterogeneity in this meta-analysis. A significant Q test (P<0.10) or I2>50% indicated heterogeneity across studies, so that the combined OR evaluated of each investigation was calculated by the random effects model, otherwise, the fixed effects model was used. Moreover, I2 values of 25, 50 and 75% were defined as low, moderate and high estimates, respectively. | Methods, Page 4 |

Page 1 of 2

| **Section/topic** | **#** | **Checklist item** | **Reported on page #** |  | |
| --- | --- | --- | --- | --- | --- |
| Risk of bias across studies | 15 | The potential publication bias was evaluated with Egger’s test and Begg’s funnel plot [20]. (References presented in the article) | Methods, Page 4-5 |  | |
| Additional analyses | 16 | Subgroup analysis was made with respect to a combination of anti-angiogenetic agents in chemotherapy regimes. In order to evaluate the stability of the outcomes, a sequential exclusion of individual studies was performed in the sensitivity analysis [19]. (References presented in the article) | Methods, Page 4 |  | |
| **RESULTS** | | |  |  | |
| Study selection | 17 | All 7 studies involving a total of 1184 patients were included in the meta-analysis, including 4 studies conducting for VEGF -2578 C/T polymorphism, 5 studies for VEGF -460 C/T, 3 studies for VEGF +405 G/C, and 5 studies for VEGF +936 C/T. The other details are presented in Figure1 (flow diagram). | Results, Page 5 |  | |
| Study characteristics | 18 | Characteristics of selected studies were listed in Table 1. | Results, Page 5 &Table 1 |  | |
| Risk of bias within studies | 19 | The results were shown in Table 2. | Results, Page 5-6 &Table 2 |  | |
| Results of individual studies | 20 | OD and 95% CI for each study are presented in Table 2. | Results, Page 5-6 & Table 2 |  | |
| Synthesis of results | 21 | In overall analysis, a significant association with responsiveness of chemotherapies in CRC has been identified in the CC vs. CA of VEGF -2578 C/A polymorphism (OR=1.40, 95% CI 1.00-1.97, P=0.05) and CC+CT vs. TT of VEGF -460 C/T polymorphism (OR=0.71, 95% CI 0.53-0.96, P=0.02). | Results, Page 5-6 |  | |
| Risk of bias across studies | 22 | Publication bias was evaluated with both visual assessment of Begg’s funnel plot and Egger’s test in the meta-analysis. As illustrated in Fig. 5, symmetrical funnel plots indicated that there was no evidence of publication bias for the meta-analysis, and the results of Begg’s test also proved the same conclusion. | Results, Page 6 |  | |
| Additional analysis | 23 | On the basis of the results in subgroup analysis, a significant association in excluding anti-angiogenetic agents subgroup was found in the comparison models of VEGF -2578 C/A polymorphism, including CC+CA vs. AA, CC vs. CA, and CA vs. AA models. Additionally, similar results were also obtained in VEGF -460 C/A polymorphism. In subgroup analysis, all positive conclusions were from excluding anti-angiogenetic agents subgroup. No association of VEGF polymorphisms with responsiveness of chemotherapies was found in the subgroup of including anti-angiogenetic agents. | Results, Page 5-6 |  | |
| **DISCUSSION** | | |  |  |  |
| Summary of evidence | 24 | In overall analysis, a significant association was found in the CC vs. CA model of VEGF -2578 C/A polymorphism, and CC+CT vs. TT model of VEGF -460 C/T polymorphism. However, no significant association was identified in other models of these two polymorphisms, and similar results were encountered in all comparison models of VEGF +405 G/C and VEGF +936 C/T polymorphisms. In subgroup analysis, a significant association was found in excluding anti-angiogenetic agents subgroup in three comparison models of VEGF -2578 C/A polymorphism, and other three genetic models of VEGF -460 C/T C/A polymorphism. | Discussion, Page 6-7 |  | |
| Limitations | 25 | Although surprising but valuable information was initially obtained in this meta-analysis, this meta-analysis was still limited due to some deficiencies. Firstly, the limited number of both studies and subjects might provide insufficient statistical power to evaluate the association between VEGF polymorphisms and responsiveness of chemotherapies. Secondly, the sources of inter-studies heterogeneity could not address for most polymorphisms. Thirdly, although there was no evident publication bias identified, potential bias might have distorted the results of the meta-analysis. Finally, relevant effect caused by other environmental factors was hard to estimate due to publication limitations or incomplete raw data. | Discussion, Page 7 |  | |
| Conclusions | 26 | This initial meta-analysis of the association between VEGF polymorphisms and responsiveness of chemotherapies in CRC was statistically more persuading than any single study. It came to a conclusion that CC vs. CA model of VEGF -2578 C/A polymorphism and CC+CT vs. TT model of VEGF -460 C/T polymorphism might be predictive factors to responsiveness of chemotherapies in CRC. However, SNPs in VEGF gene lack enough predictive ability as biomarkers to identify whether patients with CRC should add anti-angiogenetic agents into their chemotherapy regimes. | Discussion, Page 7 |  | |
| **FUNDING** | | |  |  |  |
| Funding | 27 | This work was supported by no grant. | Funding |  | |

*From:*  Moher D, Liberati A, Tetzlaff J, Altman DG, The PRISMA Group (2009). Preferred Reporting Items for Systematic Reviews and Meta-Analyses: The PRISMA Statement. PLoS Med 6(6): e1000097. doi:10.1371/journal.pmed1000097

For more information, visit: **www.prisma-statement.org**.

Page 2 of 2
