# Supplementary material for: Association between Polymorphisms in Vascular Endothelial Growth Factor Gene and Response to Chemotherapies in Colorectal Cancer: A Meta-Analysis
Source: PLoS One. 2015 May 8;10(5):e0126619. doi: 10.1371/journal.pone.0126619 (PMC4425504; doi:10.1371/journal.pone.0126619)
Supplement: S2 File — (DOC) [file pone.0126619.s002.doc]

**Meta-analysis on Genetic Association Studies Checklist | PLOS ONE**

|  | Item | Section name and paragraph number within manuscript |
| --- | --- | --- |
|  | **Introduction** |  |
| 1 | VEGF gene polymorphisms have been reported to associate with CRC through a regulation of the expression of VEGF, which has been identified to play a key role in a serious of pathologic processes involved in tumor growth and metastasis. VEGF gene polymorphisms are suggested to influence the response to chemotherapies in CRC, and it may be of great value as a potential biomarker to predict the clinical outcomes. | Introduction, paragraph 3 |
| 2 | Several single-nucleotide polymorphisms (SNPs) in VEGF gene including -2578 C/A, -460C/T, +405G/C, and +936C/T, have been focused on its relationship with the response to chemotherapies in CRC [11-17]. However, these studies showed inconclusive results, probably because the sample size enclosed in any single study is so small that it lacked inadequate evidence to demonstrate a comprehensive conclusion. (References presented in the article) | Introduction, paragraph 4 |
|  | **Methods** |  |
| 3 | All studies assessing the associations between polymorphisms in VEGF gene and response to chemotherapies in CRC were retrieved via an exhaust search of databases including Pubmed, Embase and Medline. The bibliographic search was made by two investigators using the retrieve terms as following: ("vascular endothelial growth factor a"[MeSH Terms] OR "vascular endothelial growth factor a"[All Fields] OR "vegf"[All Fields]) AND ("polymorphism, genetic"[MeSH Terms] OR ("polymorphism"[All Fields] AND "genetic"[All Fields]) OR "genetic polymorphism"[All Fields] OR "polymorphism"[All Fields])) AND (response[All Fields] OR (clinical[All Fields] AND outcome[All Fields])) AND ("colorectal neoplasms"[MeSH Terms] OR ("colorectal"[All Fields] AND "neoplasms"[All Fields]) OR "colorectal neoplasms"[All Fields] OR ("colorectal"[All Fields] AND "cancer"[All Fields]) OR "colorectal cancer"[All Fields]. Other potentially eligible studies were found by manually searching from relevant reviews and included studies. All records were updated to November 2014. Only English articles were acceptable in this meta-analysis. | Methods, Literature search |
| 4 | For an inclusion in the meta-analysis, studies had to follow such criteria: (1) studies assessing the association between polymorphisms in VEGF gene and response to chemotherapies in CRC; (2) independent prospective or retrospective association studies; (3) provided detailed data to estimate odds ratios (ORs) and according 95% confidence intervals (CIs). Studies were excluded if they were in other types of original studies such as review, meta-analysis and case reports. Moreover, studies were also not eligible for this meta-analysis for lacking critical information. | Methods, Inclusion and Exclusion criteria |
| 5 | Data extraction was conducted independently by two investigators (L. Wang and S. Ji). Inter-researcher discrepancies were settled by discussion or by a third reviewer (Z. N. Cheng). Critical data was extracted from each eligible study: first author, publication year, ethnicity, number of patients, median age, variation category, treatment modality, response criteria and genotype data. | Methods, Data extraction |
| 6 | Authors of the identified studies were contacted through E-mail if study details were required. | Methods, Data extraction |
| 7 | The adjustment of environmental effects was not conducted, because it was not a consideration in the meta-analysis. | Methods, Data extraction |
| 8 | The Cochran's Q test was used to estimate between-study heterogeneities, and I2 test was utilized to quantify the effect of heterogeneity in this meta-analysis. A significant Q test (P<0.10) or I2>50% indicated heterogeneity across studies. | Methods, Statistical analysis |
| 9 | The χ2 test was performed to appraise Hardy-Weinberg equilibrium (HWE) in the controls. | Methods, Statistical analysis |
| 10 | An application to the effects models depended on the degree of between-study heterogeneity, which was estimated by Cochran's Q test and I2 test in this meta-analysis. The heterogeneity across studies was identified by a significant Q test (P<0.10) or I2>50%, thus the random effects model was selected for the evaluation of each investigation with combined ORs. On the contrary, the fixed effects model was used for P-value of Q test bigger than 0.01 or I2<50%. | Methods, Statistical analysis |
| 11 | In order to evaluate the stability of the outcomes, a sequential exclusion of individual studies was performed in the sensitivity analysis [19]. (References presented in the article) | Methods, Statistical analysis |
| 12 | Subgroup analysis was made with respect to a combination of anti-angiogenetic agents in chemotherapy regimes. | Methods, Statistical analysis |
| 13 | ORs and 95% CIs were used to evaluate the associations of VEGF gene polymorphisms with response to chemotherapies in CRC by using Review Manager 5.3 software (provided by the Cochrane Collaboration), and a statistical significance of OR was ascertained with the P value of Z-test less than 0.05. | Methods, Statistical analysis |
|  | **Results** |  |
| 14 | The flow chart of the study selection is demonstrated in Fig. 1. | Results, Study Characteristics |
| 15 | Characteristics of selected studies were listed in Table 1. | Results, Study Characteristics |
| 16 | A summary of the meta-analysis results of the associations of VEGF gene polymorphisms with response to chemotherapies in CRC is demonstrated in Table 2. | Results,  Table 2 |
|  | **Discussion** |  |
| 17 | Some limitations in our meta-analysis was repoted in the work. | Discussion, paragraph 4 |
| 18 | In overall analysis, a significant association with responsiveness of chemotherapies in CRC has been identified in the CC vs. CA of VEGF -2578 C/A polymorphism (OR=1.40, 95% CI 1.00-1.97, P=0.05) and CC+CT vs. TT of VEGF -460 C/T polymorphism (OR=0.71, 95% CI 0.53-0.96, P=0.02). In subgroup analysis, a significant association was found in excluding anti-angiogenetic agents subgroup in three comparison models of VEGF -2578 C/A polymorphism, and other three genetic models of VEGF -460 C/T C/A polymorphism. | Discussion, paragraph 1-3 |
| 19 | This initial meta-analysis of the association between VEGF polymorphisms and responsiveness of chemotherapies in CRC was statistically more persuading than any single study. It came to a conclusion that CC vs. CA model of VEGF -2578 C/A polymorphism and CC+CT vs. TT model of VEGF -460 C/T polymorphism might be predictive factors to responsiveness of chemotherapies in CRC. However, SNPs in VEGF gene lack enough predictive ability as biomarkers to identify whether patients with CRC should add anti-angiogenetic agents into their chemotherapy regimes. In order to make a better assessment of the association between VEGF polymorphisms and responsiveness of chemotherapies in CRC, further studies conducted in standardized and unbiased ways are required. | Discussion, paragraph 5 |
