# Supplementary material for: Association between Polymorphisms in Vascular Endothelial Growth Factor Gene and Response to Chemotherapies in Colorectal Cancer: A Meta-Analysis
Source: PLoS One. 2015 May 8;10(5):e0126619. doi: 10.1371/journal.pone.0126619 (PMC4425504; doi:10.1371/journal.pone.0126619)
Supplement: S3 File — (DOC) [file pone.0126619.s003.doc]

In the literature search process, 18 full-text articles were assessed for eligibility and 11 irrelevant papers were excluded as following reasons:

Response to treatment was not investigated:

1. Dassoulas K, Gazouli M, Rizos S, Theodoropoulos G, Christoni Z, et al. (2009) Common polymorphisms in the vascular endothelial growth factor gene and colorectal cancer development, prognosis, and survival. Molecular carcinogenesis 48: 563-569.

2. Kjaer-Frifeldt S, Fredslund R, Lindebjerg J, Hansen TF, Spindler KG, et al. (2012) Prognostic importance of VEGF-A haplotype combinations in a stage II colon cancer population. Pharmacogenomics 13: 763-770.

Lacking data for odds ratio estimate:

1. Lurje G, Zhang W, Schultheis AM, Yang D, Groshen S, et al. (2008) Polymorphisms in VEGF and IL-8 predict tumor recurrence in stage III colon cancer. Annals of Oncology 19: 1734–1741.

2. Pander J, Gelderblom H, Antonini NF, Tol J, van Krieken JH, et al. (2010) Correlation of FCGR3A and EGFR germline polymorphisms with the efficacy of cetuximab in KRAS wild-type metastatic colorectal cancer. European Journal of Cancer 46: 1829-1834.

3. Loupakis F, Cremolini C, Fioravanti A, Orlandi P, Salvatore L, et al. (2011) Pharmacodynamic and pharmacogenetic angiogenesis-related markers of first-line FOLFOXIRI plus bevacizumab schedule in metastatic colorectal cancer. British journal of cancer 104: 1262-1269.

4. Gerger A, El-Khoueiry A, Zhang W, Yang D, Singh H, et al. (2011) Pharmacogenetic angiogenesis profiling for first-line Bevacizumab plus oxaliplatin-based chemotherapy in patients with metastatic colorectal cancer. Clinical Cancer Research 17: 5783-5792.

5. Formica V, Palmirotta R, Del Monte G, Savonarola A, Ludovici G, et al. (2011) Predictive value of VEGF gene polymorphisms for metastatic colorectal cancer patients receiving first-line treatment including fluorouracil, irinotecan, and bevacizumab. International journal of colorectal disease 26: 143-151.

6. Loupakis F, Ruzzo A, Salvatore L, Cremolini C, Masi G, et al. (2011) Retrospective exploratory analysis of VEGF polymorphisms in the prediction of benefit from first-line FOLFIRI plus bevacizumab in metastatic colorectal cancer. BMC cancer 11: 247-256.

7. Estevez-Garcia P, Castaño A, Martin AC, Lopez-Rios F, Iglesias J, et al. (2012) PDGFRα/β and VEGFR2 polymorphisms in colorectal cancer: Incidence and implications in clinical outcome. BMC cancer 12: 514-524.

8. Hsieh YY, Tzeng CH, Chen MH, Chen PM, Wang WS (2012) Epidermal growth factor receptor R521K polymorphism shows favorable outcomes in KRAS wild‐type colorectal cancer patients treated with cetuximab‐based chemotherapy. Cancer science 103: 791-796.

9. Price TJ, Hardingham JE, Lee CK, Townsend AR, Wrin JW, et al. (2013) Prognostic impact and the relevance of PTEN copy number alterations in patients with advanced colorectal cancer (CRC) receiving bevacizumab. Cancer medicine 2: 277-285.
